# Endocannabinoids Control Platelet Activation and Limit Aggregate Formation under Flow

**DOI:** 10.1371/journal.pone.0108282

**Published:** 2014-09-29

**Authors:** Valentina De Angelis, Arnold C. Koekman, Cees Weeterings, Mark Roest, Philip G. de Groot, Eszter Herczenik, Coen Maas

**Affiliations:** Department of Clinical Chemistry and Haematology, Utrecht University Medical Center, Utrecht, The Netherlands; University of Leuven, Belgium

## Abstract

**Background:**

The endocannabinoid system has previously been implicated in the regulation of neurons and inflammatory cells. Additionally, it has been reported that endocannabinoid receptors are present on circulating platelets, but there has been conflicting evidence on their contribution to platelet function.

**Objectives:**

Our aim was to examine the role of endocannabinoids in platelet function *in vitro* and *in vivo*.

**Methods and Results:**

We studied the effects of the well-characterized endogenous endocannabinoid anandamide on platelet aggregation in suspension, α-granule release, calcium mobilization, Syk phosphorylation, as well as platelet spreading and aggregate formation under flow. Anandamide inhibits platelet aggregation and α-granule release by collagen, collagen-derived peptide CRP-XL, ADP, arachidonic acid and thromboxane A2 analogue U46619. However, activation via thrombin receptor PAR-1 stays largely unaffected. Calcium mobilization is significantly impaired when platelets are stimulated with collagen or CRP-XL, but remains normal in the presence of the other agonists. In line with this finding, we found that anandamide prevents collagen-induced Syk phosphorylation. Furthermore, anandamide-treated platelets exhibit reduced spreading on immobilized fibrinogen, have a decreased capacity for binding fibrinogen in solution and show perturbed platelet aggregate formation under flow over collagen. Finally, we investigated the influence of *Cannabis sativa* consumption by human volunteers on platelet activation. Similar to our *in vitro* findings with anandamide, *ex vivo* collagen-induced platelet aggregation and aggregate formation on immobilized collagen under flow were impaired in whole blood of donors that had consumed *Cannabis sativa*.

**Conclusions:**

Endocannabinoid receptor agonists reduce platelet activation and aggregate formation both *in vitro* and *ex vivo* after *Cannabis sativa* consumption. Further elucidation of this novel regulatory mechanism for platelet function may prove beneficial in the search for new antithrombotic therapies.

## Introduction

Thrombosis is a worldwide leading cause of death and morbidity, caused by the formation of intravascular blood clots. It is associated with a variety of conditions, including hypercholesterolemia and diabetes mellitus type 2, in which hyperresponsiveness of platelets is a common feature [1], [2]. Currently, thrombosis is treated by administration of inhibitors of cyclooxygenase (i.e. aspirin), or alternatively, by platelet receptor antagonists, such as clopidogrel. However, these treatment modalities are associated with clinical side effects (e.g. aspirin-related gastrointestinal bleeding) and cardiovascular patients show variable responsiveness to these therapies. With the worldwide increasing incidence of cardiovascular disease, there is a correspondingly growing need for alternative therapeutic strategies to safely target pathological platelet activation.

The endocannabinoid system consists of two cannabinoid receptors (CBs), CB_1_ and CB_2_, both of which have been identified on human platelets [3]. These receptors are stimulated by endogenous ligands (endocannabinoids), but also by exogenous analogues [4]–[6]. For instance, Δ^9^-Tetrahydrocannabinol (THC), derived from *Cannabis sativa* activates endocannabinoid receptors on neurons, thereby inducing psychoactive effects [7]. Endocannabinoids are lipophilic substances synthesized from phospholipid precursors. They were originally identified as neurotransmitters and shown to be involved in the processing of information [8], pain [9], motor activity [10], blood pressure regulation [11] and immune responses [5]. Anandamide (N-arachidonylethanolamine) is one of the best-characterized endocannabinoids. It interacts both with CB_1_ and CB_2_ receptors and is present in the bone marrow microenvironment, where it regulates hematopoietic cells [12], [13]. Anandamide is a short-lived molecule, which is rapidly degraded by fatty acid amide hydrolase (FAAH). Also this enzyme has been found present in human platelets [14].

Since platelets contain these prominent components of the endocannabinoid system, the possibility exists that endocannabinoids can modulate platelet function. Several previous studies have reported controversial results about the effect of cannabinoids on platelet aggregation and –survival [14]–[18]. We here aimed to delineate the effects of anandamide on platelet function in a variety of assays, including platelet aggregation under flow. Moreover, we studied platelet function in whole blood of volunteers that had been exposed to *Cannabis sativa* for a prolonged period of time. We here report that anandamide reduces platelet responsiveness, and accordingly, lowers platelet aggregation in suspension, as well as under flow.

## Materials and Methods

### Materials

Anandamide (Arachidonylethanolamide; Sigma-Aldrich, St Louis, MO) was dissolved in dimethylsulfoxide (DMSO; Merck Chemicals Internationals, Darmstadt, Germany) and stored at −20°C. Thrombin receptor activating peptide (TRAP; SFLLRN-trifluoroacetate salt) specific for proteinase-activated receptor (PAR-1) was purchased from Bachem (Bubendorf, Switzerland), cross-linked collagen related peptide (CRP-XL) was a generous gift from Prof. dr. R. Farndale (Cambridge, UK). Horm Collagen (Kollagenreagens Horm suspension) was purchased by Nycomed (Linz, Austria); human α-thrombin and human fibrinogen from Kordia (Leiden, The Netherlands); H-D-Phe-Pro-Arg-chloromethylketone (PPACK) from Santa Cruz Biotechnology (Santa Cruz, CA); pentasaccharide (Arixtra) from GlaxoSmithKline (Greenford, UK); ADP from Sigma (Saint Louis, MO). U46619 was purchased from Cayman Chemical (Ann Arbor, MI). URB597 was purchased from Enzo Life Science GMbH (Lörrach, Germany) and indomethacin from Sigma (Saint Louis, MO). Glass coverslips for perfusion studies were from Menzel Glaser (Braunschweig, Germany). Human serum albumin (Immuno fraction V) was purchased from MP Biomedicals, Amsterdam, The Netherlands.

### Blood collection and the preparation of platelet suspensions and reconstituted blood

The local Medical Ethical Committee of University Medical Center Utrecht approved the drawing of blood for *ex vivo* research purposes, including those of this study. All donors had provided written informed consent and remained anonymous throughout the study. Blood was drawn by venipuncture from the antecubital vein of healthy donors or self-reported *Cannabis sativa* consumers [19] and collected into Vacuette tubes (Greiner, Kremsmünster, Austria) with 3.2% (m/v) trisodium citrate. In our study, we defined persons that self-reportedly used *Cannabis sativa* >4 times per week as cannabis consumers. No medication use was allowed. For controls, we included donors that had self-reportedly not consumed *Cannabis sativa* or other drug substances for at least 10 days before the experiments. Platelets were isolated as previously described. Reconstituted blood was prepared with washed platelets resuspended in 4 mM KCl, 124 mM NaCl, 20 mM NaHCO_3_, 2 mM Na_2_SO_4_, 1.5 mM MgCl_2_, 5 mM CaCl_2_, 5 mM D-glucose, 4% HSA at pH 7.3 to a platelet count of 3.33×10^11^ platelets/L. Red blood cells were isolated by centrifugation at 2,000×g for 10 minutes. The supernatant plasma was removed and the pellet was resuspended, washed twice by centrifugation for 5 minutes at 1,700×g and packed by centrifuging at 2,200×g for 12 minutes in 0.9% NaCl and 5 mM D-glucose. Red blood cells were added to the washed platelet suspension to obtain a 40% hematocrit with a platelet count of 2×10^11^ platelets/L. All cell counts were routinely determined on a CellDyn 1800 (Abbott, Hoofddorp, The Netherlands).

### Platelet aggregometry

Platelet-rich plasma (PRP) or washed platelets that had been pretreated with anandamide or vehicle (for 40 minutes unless indicated otherwise) were monitored in an optical aggregometer (Chrono-Log Corporation, Haverford, PA) at 37°C with a stirring speed of 900 revolutions per minute. Changes in light transmission in the platelet suspension were recorded for 20 minutes.

### Flow cytometric determination of platelet P-selectin expression and glycoprotein IIb/IIIa activation

Platelet responsiveness to a concentration series of CRP-XL or TRAP was determined through binding of PE-conjugated mouse anti-human P-selectin IgG (1∶25 in-assay dilution; BD Pharmingen, San Diego, CA) and 30 µg/mL Alexa-488-labeled fibrinogen (Invitrogen, San Diego, Ca). The platelet activation assay was carried out in a total volume of 55 µL by addition of 5 µL washed platelets, pretreated with anandamide or vehicle for 40 minutes. Next, the platelets were stimulated with CRP-XL, TRAP or U46619 at the indicated concentrations for 20 minutes. Finally, the samples were fixed with 500 µL 0.2% formyl saline (0.2% formaldehyde, 0,9% NaCl) and kept at room temperature until analyses on a FACSCalibur flow cytometer (Beckton Dickinson, Breda, the Netherlands).

### Platelet aggregate formation under flow

Perfusions were carried out in a single-pass perfusion chamber according to established methods that were developed in-house [20]. In brief, 24×50 mm glass coverslips were coated with 100 µg/mL type I collagen (Horm; Nycomed, Linz, Austria) or 100 µg/mL human fibrinogen for 90 minutes at RT and subsequently blocked overnight with 1% HSA at 4°C. Reconstituted blood or whole blood was perfused over collagen-coated coverslips at a shear rate of 1600 s^−1^ for 5 minutes. Washed platelets were perfused over fibrinogen-coated coverslips for 20 minutes at a constant flow rate of 25 s^−1^. Whole blood perfusion experiments were performed in the presence of 50 mM PPACK and 4300 U/mL pentasaccharide. Platelet adhesion and aggregate formation were continuously monitored using differential interference contrast microscopy with a Carl Zeiss AxioObserver Z1 and recorded through AxioCam MRm using Carl Zeiss AxioVision imaging software (Carl Zeiss MicroImaging GmbH, Gottingen, Germany) with a frequency of 1 frame/sec. A Zeiss 100x/1.3 EC PlanNeoFluar oil immersion lens was used for analysis on fibrinogen surfaces and Zeiss 40x/0.75 M27 for collagen surfaces.

### Ca^2*+*^ mobilization

PRP was incubated with 3 µM Fura 2-AM (45 minutes, 37°C, light-protected). After incubation, PRP was acidified with ACD to pH 6.5, centrifuged again (330×*g*, 15 minutes, 20°C), and resuspended in HEPES/Tyrode buffer (145 mM NaCl, 5 mM KCl, 0.5 mM Na_2_HPO_4_, 1 mM MgSO_4_, 10 mM HEPES, pH 7.25) containing 5 mM D-glucose. The final platelet concentration was adjusted to 2.0×10^11^ platelets/L. Fura-2 fluorescence was recorded in 1.0-mL aliquots of platelet suspension without additional Ca^2+^ at 20°C in a F-7000 fluorescence spectrophotometer (Hitachi Ltd., Tokyo, Japan) with excitation wavelengths of 340 and 380 nm and emission at 510 nm. Changes in cytosolic [Ca^2+^] levels were monitored using the Fura-2 fluorescence ratio and calibrated on non-activated control samples, as well as EDTA (F_min_) and digitonin-treated (F_max_) samples for reference purposes. To investigate the influence of anandamide on calcium mobilization, platelets were pre-incubated at 37°C for 40 minutes with 50 µM anandamide and subsequently stimulated by collagen, CRP-XL, ADP, U46619, TRAP or human α-thrombin during measurements.

### Immunoprecipitation and western blotting

Platelets were stirred at 900 rpm and pre- incubated at 37°C for 40 minutes with and without 50 µM anandamide and subsequently stimulated with either 1 µg/mL collagen or 0.1 U/mL human α-thrombin for 5 or 10 minutes. Platelet suspensions (450 µL of 2×10^11^ platelets/L) were lysed in RIPA lysis buffer (1% m/v Nonidet P-40, 0.5% m/v Octylglucoside, 0.1% SDS, 5 mM EDTA), supplemented with 1 mM orthovanadate for 15 minutes on ice to prevent dephosphorylation after lysis. Lysates were mixed with 55 µL (10% vol/vol) protein G sepharose that had been pre-adsorbed with 1 mg/mL anti–Syk IgG (sc573; Santa Cruz Biotechnology, Santa Cruz, CA,) for 60 minutes at 4°C under rotation. Proteins were separated by sodium dodecylsulfate-polyacrylamide gel electrophoresis and subjected to western blotting. After incubating with blocking buffer (Odyssey; LI-COR, Lincoln, NE), 1 mg/mL anti-Syk IgG or anti-phosphotyrosine IgG (4G10; Upstate Biotechnology, Lake Placid, NY) was added to the membranes and finally the protein bands were visualized with an Odyssey Imaging system (LI-COR Biosciences, Lincoln, NE). Quantification was performed with Image-J software (NIH, Bethesda, MD). Possible lane-to-lane loading variation was corrected by normalization to the total amount immuno-precipitated Syk protein.

### Data analysis

FACS data samples were analyzed on a FACS Canto II flow cytometer from BD Biosciences (Franklin Lakes, NJ). The spreading behaviour of individual platelets was kinetically analyzed with MacBiophotonics ImageJ software 1.45s for Windows (Wayne Rasband, National Institute of Health). At fixed timepoints, platelet contours were digitally drawn and the area within was quantified. Dose-dependency inhibition studies were statistically analysed by non-parametric one-way ANOVA, followed by Kruskal-Wallis post-testing for inter-group differences. Individual inter-group comparisons and western blotting densitometry experiments were compared by Mann-Whitney U tests. Values are expressed as mean +/− SD. Asterisks indicate *p<0.05; **p<0.01; ***p<0.001; ****p<0.0001.

## Results

### Anandamide inhibits platelet aggregation in a concentration- and time-dependent manner

In an initial series of experiments, we aimed to characterize the effects of anandamide on platelet aggregation. Hereto, we first pretreated washed human platelets with anandamide and subsequently monitored the aggregation responses to various platelet agonists. Platelet aggregation can proceed through several pathways: type I collagen directly activates platelets through a glycoprotein VI (GPVI)-dependent pathway, which can also be triggered by the soluble crosslinked collagen-related peptide CRP-XL. Adenosine diphosphate (ADP) induces activation through the receptor P_2_Y_12_. Arachidonic acid (AA) indirectly triggers aggregation through formation of thromboxane A2 (TxA2; represented by a stable analogue U46619 in our experiments). Finally, thrombin activates the protease activated receptor 1 pathway (PAR-1), which can also be triggered by thrombin receptor activating peptide (TRAP).

**Figure 1:**
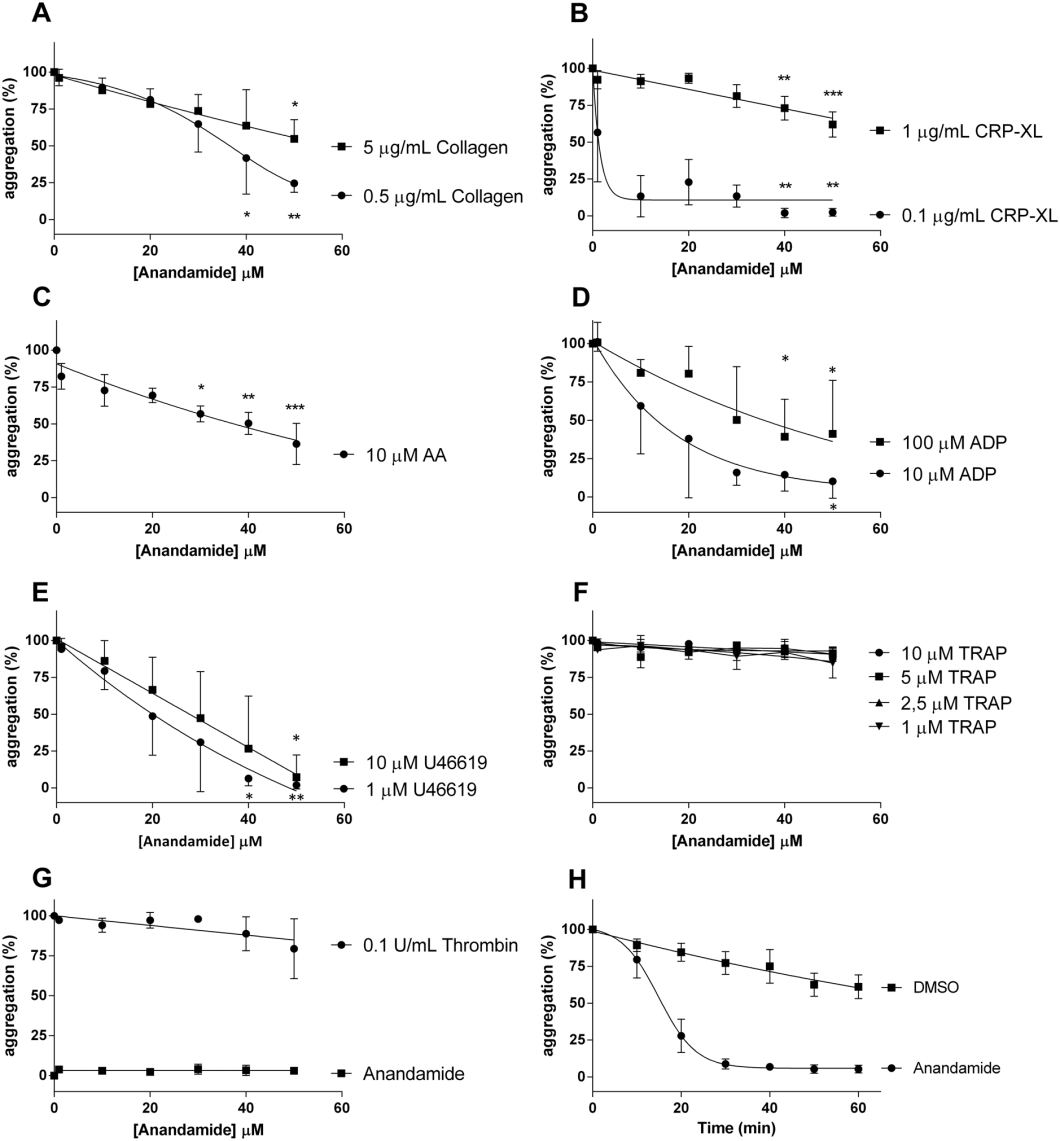
The endocannabinoid anandamide inhibits platelet aggregation. Washed platelets were exposed to a concentration series of anandamide for 40 minutes at 37°C. Subsequently, they were stimulated with 0.5 or 5 µg/mL collagen (A; representative aggregation curves of 0.5 µg/mL collagen stimulation are shown in Figure S1A); 0.1 or 1 µg/mL CRP-XL (B; representative aggregation curves of 0.1 µg/mL CRP-XL are shown in Figure S1B); 10 µM Arachidonic Acid, (AA) (C; representative aggregation curves are shown in Figure S1C); 10 or 100 µM ADP (D; representative curves of 10 µM are shown in Figure S1D); 1 or 10 µM U46619 (E; representative aggregation curves of 1 µM U46619 are shown in Figure S1E); 1, 2.5, 5 or 10 µM TRAP (F; representative aggregation curves of 2.5 µM are shown in Figure S1F), 0.1 U/mL thrombin or anandamide only (G; representative aggregation curves are shown in Figure S1G). Data were normalized to 100% platelet aggregation obtained with the agonists alone in the presence of vehicle. The time-dependence of anandamide inhibition was investigated by incubating washed platelets with 50 µM anandamide or vehicle (DMSO) at 37°C in a time series and subsequently stimulated with 0.5 µg/mL collagen (H). Data are mean ± SD and show 3 or more individual experiments.

In a series of aggregation experiments, we found that increasing concentrations of anandamide (from 1 to 50 µM) progressively inhibit platelet aggregation when triggered by 0.5 or 5 µg/mL type I collagen (Fig. 1A representative aggregation curves are shown in Figure S1A); 0.1 or 1 µg/mL CRP-XL (Fig. 1B, Figure S1B). Similarly, anandamide inhibits platelet aggregation by 10 µM Arachidonic Acid, (AA, Fig. 1C, Figure S1C); 10 or 100 µM ADP (Fig. 1D, Figure S1D) as well as by 1 or 10 µM U46619 (Fig. 1E, Figure S1E). Although these findings suggested that anandamide acts as a general platelet antagonist, we found that it does not inhibit platelet aggregation triggered by TRAP (1–10 µM, Fig. 1F, Figure S1F) or by 0.1 U/mL thrombin (Fig. 1G, Figure S1G). Furthermore, we found that 1–50 µM anandamide does not independently induce platelet aggregation (Fig. 1G, Figure S1G). Our aggregation experiments together suggest that anandamide interferes with platelet aggregation through the GPVI-, ADP- and AA/TxA_2_-pathway; but not when platelets are activated through the thrombin receptor PAR-1 pathway.

We next investigated in what time-frame anandamide started to influence platelet aggregation. Hereto, we incubated washed platelets with a fixed concentration of anandamide (50 µM) in a time series and subsequently stimulated them with 0.5 µg/mL collagen (Fig. 1H). When platelets are preincubated with anandamide for 10 minutes prior to activation, a modest inhibitory effect is observed (∼80% residual platelet aggregation). However, after a preincubation of 30 minutes or more, collagen-induced platelet aggregation is maximally inhibited by anandamide (<8% residual aggregation). Together, these results show that the endocannabinoid anandamide inhibits platelet aggregation in a concentration and time-dependent manner.

### Anandamide inhibits α-granule secretion and limits platelet aggregate formation

**Figure 2:**
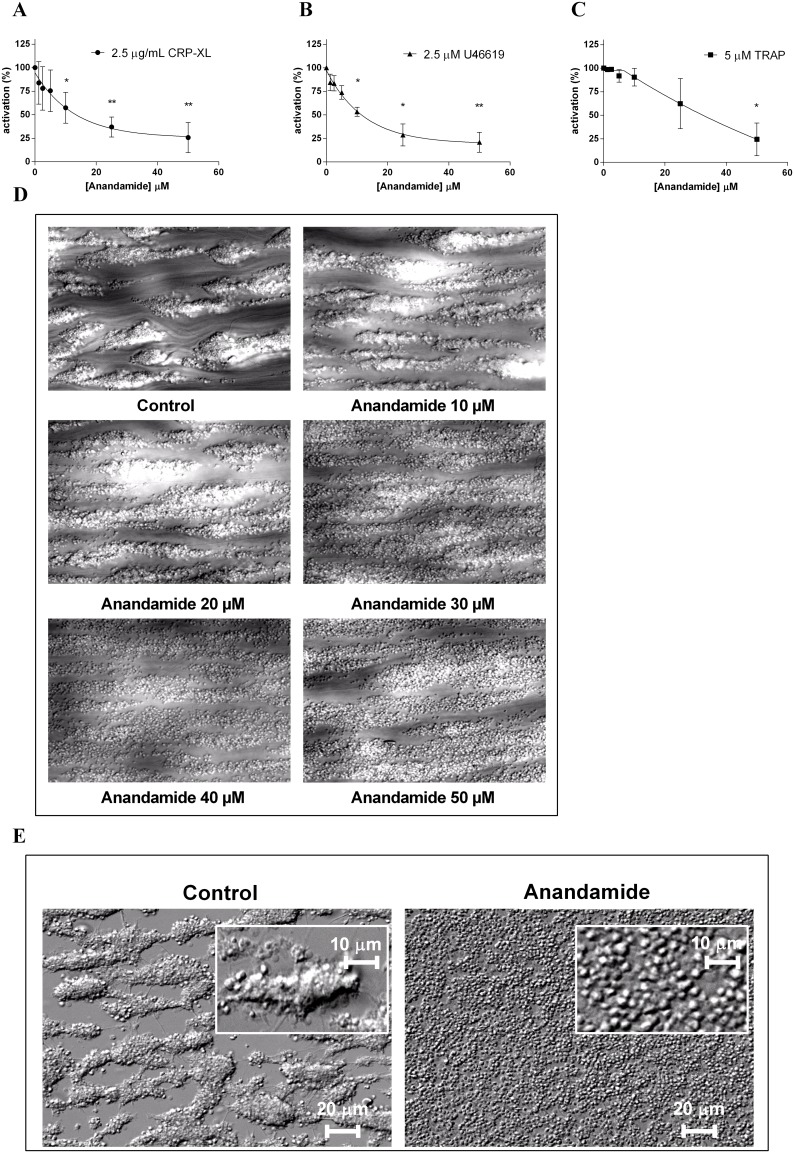
Anandamide inhibits platelet α-granule secretion and limits platelet aggregate formation. Platelet α-granule release was studied by flow cytometric analysis of platelet P-selectin externalization. Washed platelets from healthy human donors were pre-exposed to a concentration series of anandamide for 40 min at 37°C and subsequently stimulated with 2.5 µg/mL CRP-XL (A), 2.5 µM U46619 (B) or 5 µM TRAP (C). Data were normalized to the agonist-induced P-selectin expression of vehicle-pretreated control platelets. Reconstituted blood, which was preincubated with anandamide, was perfused over a collagen-coated surface at a shear rate of 1600 s^−1^ and snapshots were taken after 5 minutes (D). Subsequently, cover slips were rinsed with buffer for 1 minute to investigate aggregate stability (E). The left panel shows vehicle control, the right panel indicates a perfusion in the presence of 50 µM anandamide. Data are shown as mean ± SD and represent 3 or more individual experiments.

In order to elucidate what mechanisms are involved in the inhibition of platelet aggregation by anandamide, we first examined α-granule release during platelet activation by determining P-selectin externalization by flow cytometry. When platelets are stimulated with either 2.5 µg/mL CRP-XL (Fig. 2A), 2.5 µM U46619 (Fig. 2B) or 5 uM TRAP (Fig. 2C), all platelets externalize P-selectin. In comparison, unstimulated platelets retain P-selectin intracellularly (not shown). Anandamide inhibits P-selectin expression by platelets in a dose-dependent fashion when they are stimulated with CRP-XL, U46619, or TRAP.

We next analyzed whether the exposure of platelets to anandamide also affects aggregate buildup under flow. When untreated reconstituted blood is perfused over collagen at an arterial shear rate (1600 s^−1^), the adhesion of untreated platelets and subsequent aggregation occurs within 5 minutes (Fig. 2D, indicated as “control”:). In contrast, anandamide-treated platelets adhere to the collagen surface but are unable to form normal aggregates. In line with our earlier aggregometry experiments (Fig. 1), we found that this inhibitory effect of anandamide is dose-dependent (Fig. 2D). At lower anandamide concentrations (10 and 20 µM), platelet aggregate formation is comparable to that of the untreated platelets. However, at higher doses (30–50 µM anandamide), platelet aggregation becomes perturbed to an equal extent. We observed little difference at anandamide concentrations over 20 µM, which may be caused to the disaggregating effect of shear flow, which was not present in our earlier aggregometry studies.

To study the platelet-platelet interactions and aggregate stability, we subsequently perfused buffer for one minute over preformed aggregates. In contrast to aggregates of untreated platelets, this extra washing step further destabilized platelet clots that previously received high doses of anandamide and induced the formation of a disperse monolayer of discoid-shaped platelets on the collagen surface (Fig. 2E; the images in the right panel show platelets that were preincubated with 50 µM anandamide). These experiments indicate that anandamide modulates platelet reactivity by limiting α-granule secretion and reducing platelet aggregate formation under flow.

### Anandamide inhibits platelet spreading on fibrinogen by limiting glycoprotein IIb/IIIa activation

**Figure 3:**
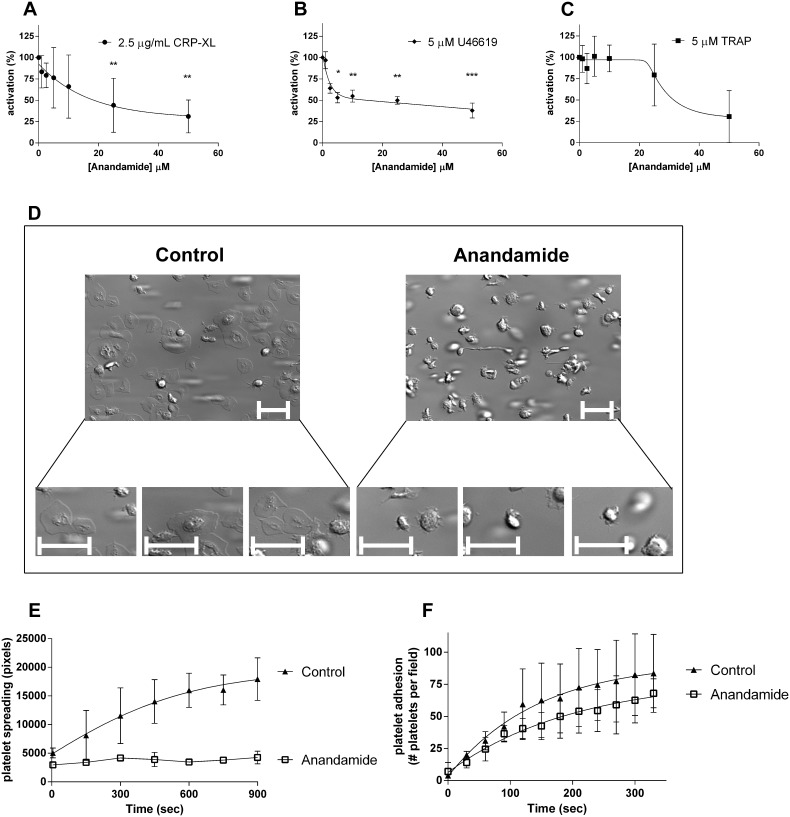
Anandamide reduces glycoprotein IIb/IIIa activation and inhibits platelet spreading. Washed platelets from healthy human donors were exposed to a concentration series (0–50 µM) of anandamide for 40 minutes at 37°C and subsequently stimulated with 2.5 µg/mL CRP-XL (A), 2.5 µM U46619 (B) or 5 µM TRAP (C). Platelet GPIIb/IIIa activation was studied by flow cytometry. Data were normalized to the agonist-induced GPIIb/IIIa activation of vehicle-pretreated control platelets. Washed platelets were preincubated with 50 µM anandamide or vehicle for 40 minutes at 37°C and perfused over immobilized fibrinogen- at a shear rate of 25 s^−1^. Subsequently, differential interference contrast microscopy images were taken (D; representative images after 15 minutes of perfusion, scale bars represent 10 µm). Quantification of the spreading behaviour of individual platelets on immobilized fibrinogen (E; spreading was quantified for 3 individual platelets per separate experiment). Quantification of the amount of adhering platelets per field (F). Data represent mean and standard deviation (SD) of 3 or more individual experiments.

We next investigated the effects of anandamide on the activation of glycoprotein IIb/IIIa **(**GPIIb/IIIa) on platelets by determining the amount of platelet-bound FITC-labeled fibrinogen by FACS. We found that anandamide inhibits GPIIb/IIIa activation, when triggered by 2.5 µg/mL CRP-XL (Fig. 3A) or 5 µM U46619 (Fig. 3B). However, only high concentrations (50 µM) of anandamide were able to reduce TRAP-induced fibrinogen binding activation (Fig. 3C). These data indicate that anandamide interferes with the fibrinogen-binding capacity of platelets, but apparently not sufficiently to inhibit platelet aggregation in our earlier aggregometry experiments (Fig. 1). These findings are in agreement with previous studies that reported that TRAP-induced platelet aggregation can occur partially independently of GPIIb/IIIa [21], [22].

The adhesion and spreading of platelets on immobilized fibrinogen at a low shear rate (25 s^−1^) is dependent on GPIIb/IIIa. We next investigated the effects of anandamide on this process. While control platelets adhere to fibrinogen and gradually spread to cover the surface (Fig. 3D; quantification of the spreading behaviour of individual platelets in Fig. 3E), anandamide-treated platelets adhere to fibrinogen in comparable quantities (quantified in Fig. 3F) but are unable to spread normally (Fig. 3D, E).

### Anandamide blocks collagen-induced platelet signaling

**Figure 4:**
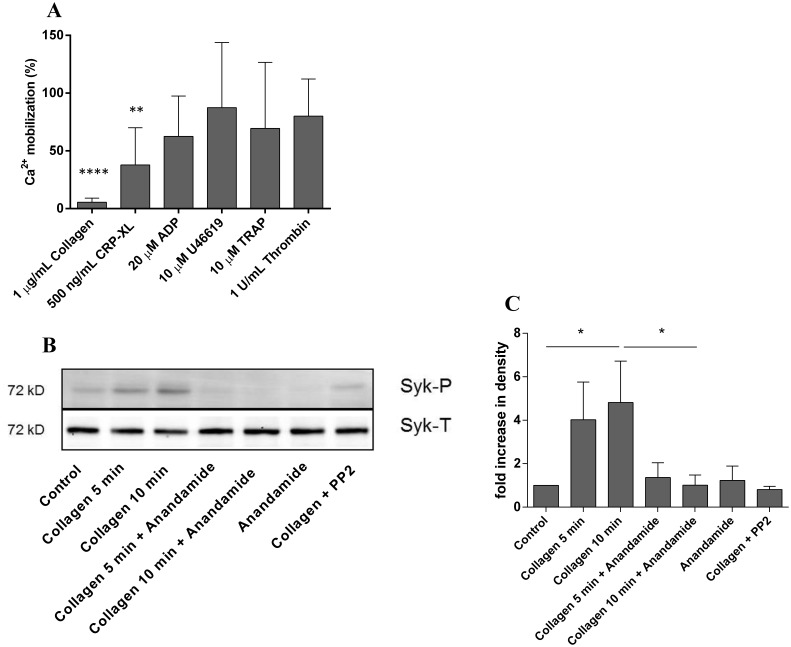
Anandamide inhibits glycoprotein VI-dependent calcium mobilization and Syk-phosphorylation. Washed platelets were preincubated with 50 µM anandamide or vehicle for 40 minutes at 37°C. Subsequently, they were exposed to 1 µg/mL collagen, 500 ng/mL CRP-XL, 20 µM ADP, 10 µM U46619, 10 µM TRAP or 1 U/mL thrombin and calcium mobilization was monitored. The (residual) calcium mobilization in the presence of anandamide is expressed as a percentage of uninhibited vehicle control in the presence of the same platelet agonist (A). Western blot analysis of collagen-induced Syk phosphorylation in the presence of vehicle, 50 µM anandamide or control Syk phosphorylation inhibitor PP2 (B; upper lanes indicate phosphorylated Syk (Syk-P), the lower lanes indicate total Syk antigen as a loading control (Syk-T)). Densitometric quantification of collagen-induced Syk-phosphorylation in the presence or absence of anandamide or PP2 (C; n = 4: data are expressed as mean +/− SD).

We earlier found that anandamide inhibits platelet aggregation induced by collagen and CRP-XL (Fig. 1), which requires signaling events through GPVI and Ca^2+^ mobilization. Phosphorylation of spleen tyrosine kinase (Syk) by Src-family kinases represents a key intermediate step in this pathway. The intracellular events involved in the suppressing effect of anandamide were first studied by investigating the intracellular Ca^2+^ levels in platelets after stimulation with collagen, CRP-XL, as well as with ADP, U46619, TRAP and thrombin (Fig. 4A). We found that the mobilization of Ca^2+^ in platelets that are activated by collagen and CRP-XL is significantly reduced in the presence of anandamide (Fig. 4A), while Ca^2+^ mobilization remained unaffected when platelets are activated by ADP, U46619, TRAP or thrombin (Fig. 4A). Moreover, when analyzing the phosphorylation status of Syk in collagen-stimulated platelets, the characteristic time-dependent increase in phosphorylation was absent in the presence of anandamide, as well as in the presence of control inhibitor PP2 (a Src-family kinase inhibitor; representative western blot shown in Fig. 4B and densitometric quantification of repeated experiments in Fig. 4C). Our results show that anandamide inhibits Ca^2+^ mobilization and prevents Syk phosphorylation in platelets that are stimulated by collagen, which suggest that anandamide has the capacity to block specific elements of the collagen pathway.

### Platelet inhibition by anandamide is not dependent on platelet preactivation

**Figure 5:**
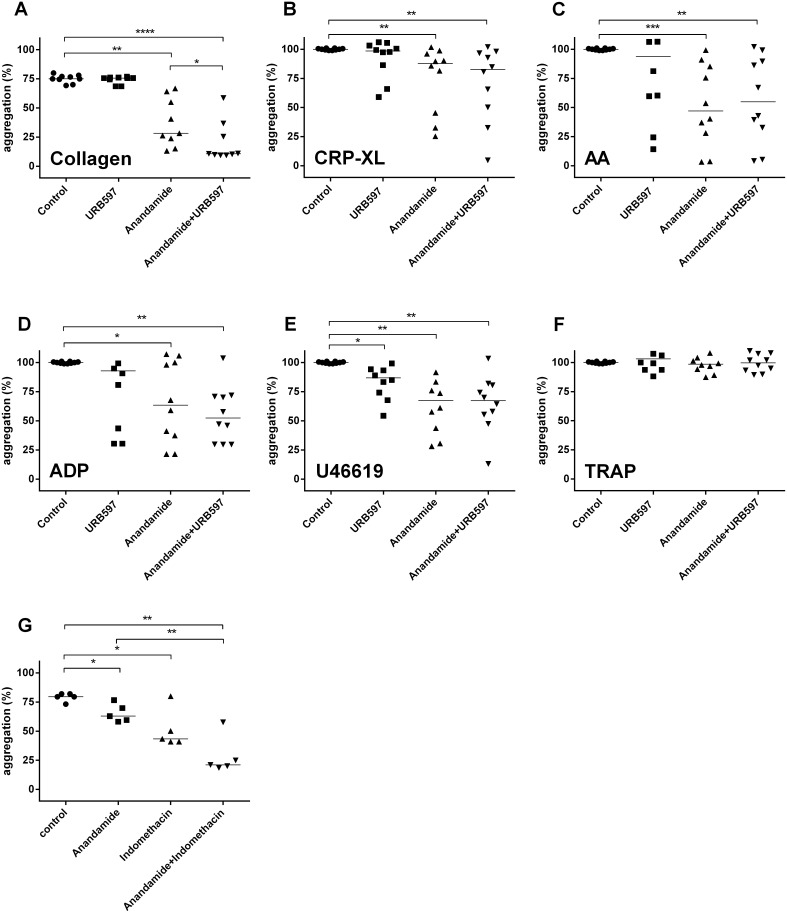
The inhibitory effect of anandamide on platelet activation is not dependent on platelet preactivation. Washed platelets were pretreated with 25 µM FAAH-inhibitor URB597. Subsequently, platelet aggregation was induced by various agonists after a suboptimal exposure to anandamide (10 µM, preincubated for 40 minutes). 0.5 µg/mL collagen (A), 0.1 µg/mL CRP-XL (B), 10 µM AA (C), 10 µM ADP (D), 1 µM U46619 (E) or 1 µM TRAP (F). In further experiments, washed platelets were pretreated with 10 µM indomethacin, exposed to anandamide (10 µM, preincubated for 40 minutes) and stimulated with collagen (0.5 µg/mL: G).

The biological effects of anandamide *in vivo* are tightly regulated through degradation and uptake mechanisms that limit its extracellular lifetime [23]. In platelets this is amongst others mediated by fatty acid amide hydrolase (FAAH), which hydrolyzes anandamide into ethanolamine and AA. This latter molecule is a precursor of thromboxane A2 (TXA_2_), which is a well-known platelet agonist. Although our earlier aggregometry experiments suggested otherwise (Fig. 1G), the observed lowered platelet activation in the presence of anandamide could potentially be an indirect result of desensitization through TXA_2_ formation. URB597 is a selective inhibitor of FAAH that can be applied *in vivo* to prevent anandamide degradation, resulting in elevated tissue levels of anandamide and enhanced biological effects [24]. To explore the possibility that anandamide required processing by FAAH to inhibit platelet activation, we investigated the effects of URB597 on platelet inhibition by anandamide. After 20 minutes of pre-incubation with 25 µM URB597 to block FAAH, platelets were incubated with an intermediate concentration of 10 µM anandamide for an additional 40 minutes. We found that the inhibitory properties of anandamide are not negatively influenced by URB597 (Fig. 5A–F), indicating that FAAH activity is not required for platelet inhibition by anandamide. As expected, platelet activation by TRAP is unaffected by anandamide, and remains unaffected in the presence of URB597 (Fig. 5F). Furthermore, we found that URB597 significantly enhances the effects of anandamide when platelets are activated by collagen (Fig. 5A). Finally, we found that URB597 independently inhibited U46619-induced platelet aggregation (Fig. 5E), which could point to a potential contribution of FAAH to platelet activation. These experiments show that the inhibitory capacity of anandamide either slightly increases (collagen) or remains unchanged (CRP-XL, AA, ADP, U46619) when FAAH is blocked, arguing against a role for anandamide-induced platelet preactivation of platelets.

In a following experiment, we pretreated platelets with indomethacin and investigated the inhibitory potential of anandamide on collagen stimulation. Indomethacin blocks the conversion of arachidonic acid into TXA_2_, reducing platelet excitability. Accordingly, if platelet inhibition by anandamide is the result of preactivation (through the release of AA), indomethacin would reverse it. However, we found that the inhibitory effect of anandamide is persistent in the presence of indomethacin (Fig. 5G), but both inhibitors are not synergistic together. Together, these data show that the inhibitory effect of anandamide on platelets is independent of AA formation.

### Impaired platelet aggregate formation in *Cannabis sativa* consumers

**Figure 6:**
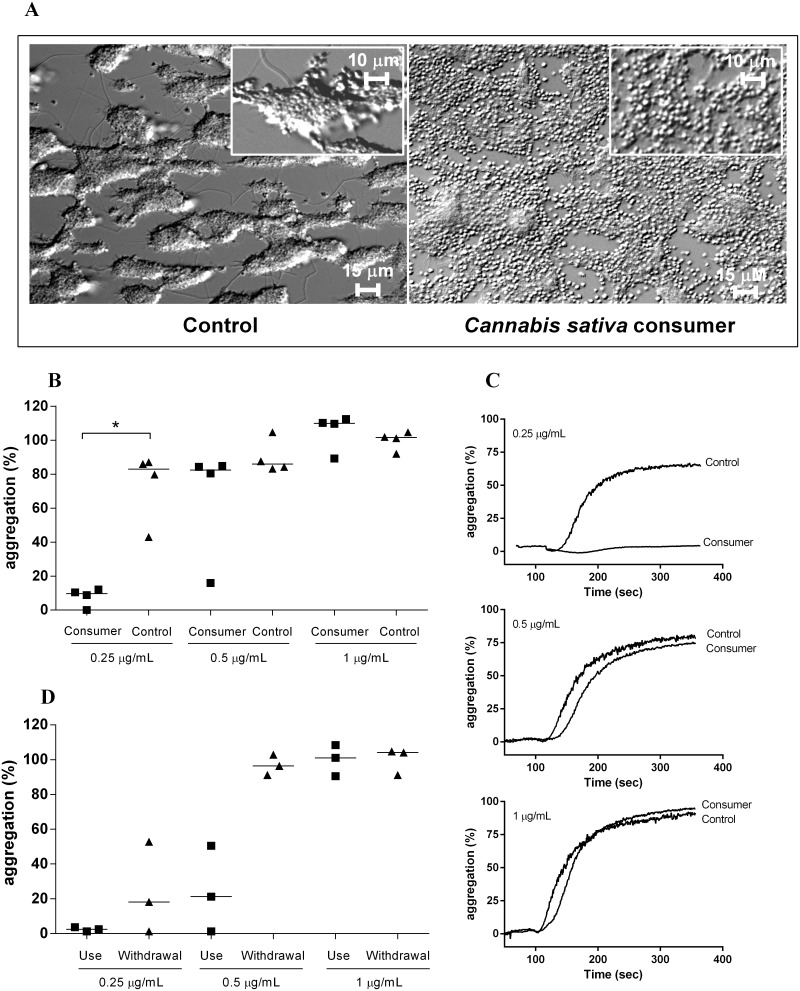
*Cannabis sativa* consumption limits platelet aggregate formation under flow and reduces platelet responsiveness to collagen. Whole blood from *Cannabis sativa* consumers (n = 4; “Consumers”) or healthy control donors (“Controls”) was perfused for 5 minutes over immobilized collagen at a shear rate of 1600 s^−1^ (A). In further experiments, collagen-induced platelet aggregation was investigated in platelet-rich plasma from these *Cannabis sativa* consumers and controls (B). Representative aggregation curves are shown in panel C. Finally, collagen-induced platelet aggregation was studied in platelet-rich plasma of three self-reported *Cannabis sativa* consumers on two separate instances: once after 10 days of daily consumption of *Cannabis sativa* (“Use”), as well as after a period of 10 days without consumption (“Withdrawal”).

THC, the psychoactive component of Cannabis sativa, functionally mimics the effects of anandamide on endocannabinoid receptors. We next investigated platelet function in citrated whole blood, donated by self-reported *Cannabis sativa* consumers. Similar to our earlier experiments (Fig. 2), we perfused whole blood over immobilized collagen at an arterial shear rate of 1600 s^−1^. In the blood of control donors, platelets adhere to the surface and subsequently form stable aggregates (Fig. 6A, left panel; Video S1 shows a perfusion experiment with a representative control donor). In contrast, we found that platelets from *Cannabis sativa* consumers initially adhere to the surface in comparable fashion, but are unable to form stable aggregates; single discoid platelets were constantly visible during the perfusion, but aggregate formation under flow appeared impaired (Fig. 6A, right panel; Video S2 shows a representative *Cannabis sativa* consumer). These findings are strikingly similar to our earlier experiments with anandamide-treated platelets in reconstituted blood (Fig. 2D, E).

Subsequently, we analyzed collagen-induced platelet aggregation in platelet-rich donor plasma from four *Cannabis sativa* consumers and four healthy donors. Similarly, we also analyzed collagen-induced platelet aggregation in another group of three donors: once after 10 days of daily *Cannabis sativa* consumption, and once after 10 days of discontinuation (i.e. not having consumed *Cannabis sativa*). We found no differences in platelet counts between *Cannabis sativa* consumers and controls (data not shown). When platelet aggregation was triggered by 0.25 µg/mL collagen (Fig. 6B; representative aggregation curves in Fig. 6C), all four donors that had consumed *Cannabis sativa,* showed diminished responsiveness compared control subjects. Similarly, the three donors that had repeatedly consumed *Cannabis sativa* appeared to be less responsive than after a period of withdrawal (Fig. 6D). However, this relation failed to reach statistical significance, presumably due to the small group size (n = 3). At higher collagen concentrations, consumers and controls responded comparably (Fig. 6B). These findings are similar to the *in vitro* effects of anandamide on platelets in our earlier experiments (Fig. 1A) and suggest that endocannabinoid-like substances reduce platelet activation and –aggregation in response to mild triggers *ex vivo*.

## Discussion

Atherothrombosis is a leading worldwide cause of death and disability. The currently available antiplatelet therapies are known for their side effects and inconsistent therapeutic efficacy. As the incidence of thrombotic disease is increasing, there is a growing need for new therapeutic strategies. The endocannabinoid system is involved in several physiological processes and the use of cannabinoids as therapeutic agents has been proposed in a variety of diseases. For these therapeutic purposes, the development of peripherally restricted and non-psychoactive pharmacological endocannabinoid analogues is warranted [25].

The influence of endocannabinoids on platelet function and thrombus formation is subject to debate: previous studies have addressed the relationship between cannabinoid receptors and platelet function with conflicting outcomes. For instance, it was previously shown that direct exposure of whole blood to THC induces platelets to externalize P-selectin [15], but platelet aggregation was not investigated in that study. In sharp contrast, other studies showed that aggregation of isolated platelets is effectively inhibited by various cannabinoids derived from *Cannabis sativa* (including THC) [17].

In the present study, we report that the endocannabinoid anandamide lowers platelet responsiveness to mildly activating triggers in a dose- and time-dependent fashion. As a result, their capacity for aggregation in suspension, as well as under flow is lowered. Our data suggest that these inhibitory effects of anandamide are related to a reduced α-granule secretion and decreased GPIIb/IIIa activation. We also found that collagen-and AA-induced platelet activation was particularly affected by anandamide, while activation through PAR-1 remains largely unaffected. This suggests that endocannabinoids may have specific intracellular targets in the affected platelet activation pathways.

To explain these findings, we hypothesize that anandamide (amongst others) interferes with the function of G-proteins in platelets. Since AA and TxA2-receptor mediated aggregations depend on G13 and Gq (Figure 1C and Figure 1), we found that these aggregations are affected by anandamide. In contrast, ADP-aggregations depend on Gi, which we found to be partially inhibited as well (Figure 1D). Finally, PAR-mediated platelet aggregation operates through all three types of G-proteins together. Our data suggests that this latter mechanism of activation is sufficient to overcome inhibition by anandamide (Figure 1F and G), which may point to a synergism between G-protein mediated pathways of platelet activation.

The levels of anandamide that were used in our studies ranged up to 50 µM. However, levels of free anandamide in citrated plasma are around 1 nM [26]. Plasma THC levels are reportedly 150 ng/mL (0.47 µM) immediately after *Cannabis sativa* consumption [27]. Since endocannabinoids are generally lipophilic in nature, they may become rapidly recruited to lipophilic milieus such as the bone marrow environment, potentially leading to elevated local concentrations (and low plasma concentrations). Finally, a recent report shows that anandamide can be safely administered intraperitoneally at 20 mg/kg to 12-week-old mice. Assuming that these mice have a circulating volume of 2 mL (and equal distribution), this would result in an overall concentration of ∼576 µM [28], which is far above the levels used in our studies. Platelet inhibition by anandamide can be explained in two opposing ways: either the platelets are actively inhibited, or alternatively, they are desensitized through (partial) preactivation. We propose that platelets are actively inhibited, rather than preactivated, based on two lines of evidence. Firstly, platelets are fully responsive to activation by TRAP and thrombin, even in the presence of high levels of anandamide (Fig. 1K). Secondly, if anandamide were to preactivate platelets, it would have to be a result of its degradation into arachidonic acid and subsequent conversion into TXA_2_. However, we observed a persistent inhibition of platelets by anandamide, even in the presence of an inhibitor of FAAH (that prevents anandamide degradation), or indomethacin that prevents TXA_2_ formation.

In our experiments, we found a remarkable similarity between our *in vitro* data and the *ex vivo* platelet function of *Cannabis sativa* consumers. In these subjects, a reduced response towards collagen was detected in aggregometry experiments. The aggregate morphology that we observed during perfusion experiments strongly resembled earlier *in vitro* findings with anandamide: round-shaped platelets that are unable to form normal aggregates. Interestingly, this aggregate phenotype is also commonly observed in patients treated with antiplatelet therapies, such as clopidogrel. However, despite impaired platelet functionality, *Cannabis sativa* consumers are not commonly known to have a bleeding phenotype. At present it is premature to conclude that anandamide or similar compounds are useful as antithrombotic agents. Further investigations are required to establish whether anandamide-like compounds can be effectively used as antithrombotics with lower side-effects than currently used antiplatelet agents.

Additionally, our findings raise an interesting question: what role does the endocannabinoid-dependent inhibitory pathway play in platelet physiology? It has been described that anandamide extends platelet viability *in vitro*, which may be useful for the *ex vivo* storage of platelet concentrates [18]. Interestingly, this mechanism could also reflect an innate ‘preservation’ pathway present in platelets, meant to prevent premature activation of platelets in the bone marrow niche. Indeed, there is evidence that the endocannabinoid system is active in the bone marrow: the cannabinoid receptor *Cb2* is expressed by hematopoietic cell lines and endocannabinoids reportedly regulate cells in the bone marrow environment [12], [13]. Based on our findings, it is attractive to think that endocannabinoids instruct platelets to be less sensitive to triggers that are likely to be present in uninjured bone marrow (i.e. collagen and arachidonic acid), while platelet responses to triggers that are formed during actual injury of the bone marrow remain intact (i.e. thrombin).

In conclusion, our results indicate that (endo)cannabinoids suppress platelet activation and aggregate formation both *in vitro* and *ex vivo*. Since our perfusion studies were performed in the absence of plasma, more detailed future studies will have to address whether the endocannabinoid system is also involved in *in vivo* thrombus formation. Similarly, the therapeutic potential for targeting pathological thrombus formation via the endocannabinoid system still remains to be determined. However, as therapeutic (endo)cannabinoid analogs are currently being developed for treatment of a multitude of diseases, it may be interesting to investigate whether these compounds also have antithrombotic properties.

## Supporting Information

Figure S1 The endocannabinoid anandamide inhibits platelet aggregation. Washed platelets were exposed to a concentration series of anandamide or vehicle for 40 minutes at 37°C. Subsequently, aggregation was induced by A) 0.5 µg/mL collagen B) 0.1 µg/mL CRP-XL C) 10 µM Arachidonic Acid, (AA) D) 10 µM ADP, E) 1 µM U46619 F) 2.5 µM TRAP, or G) 0.1 U/mL thrombin or anandamide only. Shown aggregation data are representative for 3 or more individual experiments.
(TIF)

Video S1 **Control subject.** Shown at 10 frames per second.
(MOV)

Video S2 ***Cannabis sativa*** **consumer.** Shown at 10 frames per second.
(MOV)
